# Chemical Compositionand Anti-acetyl cholinesterase Activity of Flower Essential Oils of Artemisiaannuaat Different Flowering Stage

**Published:** 2011

**Authors:** Zhengwen Yu, Bochu Wang, Fumei Yang, Qianyun Sun, Zhannan Yang, Liancai Zhu

**Affiliations:** a Bioengineering College of Chongqing University and Drug Screening Lab, The Key Laboratory of Chemistry for Natural Products of Guizhou Province and Chinese Academy of Science.; b School of Life Sciences of Guizhou Normal University, Guiyang, Guizhou, 550001, P.R.China.; c Drug Screening Lab, Key Laboratory of Chemistry for Natural Products of Guizhou Province and Chinese Academy of Science, 550001, P.R.China.; d Guizhou Provincial Laboratory for Mountainous Environment (GLE), Guizhou Normal University, Guiyang, Guizhou 550001, P.R.China.

**Keywords:** *Artemisia annua*, Essential oils, Anti-acetylcholinesterase activity

## Abstract

The chemical composition of the essential oils of flower at the pre-flowering, full-flowering and post-flowering stage of *A. annua* was analyzed by GC and GC/MS and sixty-two components were identified. The main compounds in the pre-flowering oil were *β*-myrcene (37.71%), 1, 8-cineole (16.11%) and camphor (14.97%). The full-flowering oil contained predominantly caryophyllene (19.4%), germacrene D (18.1%), camphor (15.84%), 1, 8-cineole (10.6%) and (Z)-*β*-farnesene (9.43%). The major constituents identified in the post-flowering oil were camphor (16.62%), caryophyllene (16.27%), *β*-caryophyllene oxide (15.84%), *β*-farnesene (9.05%) and (-)-spathulenol (7.21%).

The variety of anti-AChE activity of flower oil of *A. annua* at three flowering stage might be a result of the variety of the content and interaction of those terpenoids with anti-AChE activity. The greatest acetylcholinesterase inhibitory activity (IC_50_ = 0.13 ± 0.02 mg mL^-1^) was exhibited by the essential oil of flower of *A. annua* at post-flowering stage.

## Introduction

*Artemisia annua* L. is an annual native herb of China, where it has been used in the treatment of fever and malaria for many centuries. Many secondary metabolites of terpene peroxides were isolated from the plant, such as artemisia ketone, artemisinic alcohol, arteannuin B and myrcene hydroperoxide (1, 2). The most famous terpene peroxide is artemisinin with a chemical structure of amorphane-type sesquiterpene endoperoxide, and it has become an important plant-derived compound in the treatment of the chloroquine-resistant and cerebral malarias (3). The essential oils, another important composition of *A. annua*, have been subjected to extensive phytochemicals and pharmacological activity studies. The percentage of artemisia ketone, 1, 8-cineole, camphor, borneol, germacrene D and *α*-pinene was reported to vary from 0.0-80.9%, 0-31.5%, 0-27.5%, 0-20.0%, 0-18.9% and 0-16.0%, respectively. Other major reported components were artemisia alcohol, *β*-pinene, carvacrol, thymol, myrcene, limonene, camphene, copaene, *β*-caryophyllene, *α*-terpineol, *α*-elemene,*β*-elemene and *γ*-elemene, sabinene, *α*-guaiene, caryophyllene, caryophyllene oxide, and so on. The biological activities reported for the compounds isolated from *A. annua* essential oils, are antibacterial, anti-inflammatory, angiotensin converting enzyme inhibitory, plant growth regulatory, cytokinin-like and antitumor (4).

Alzheimer is a progressive degenerative neurologic disorder resulting in impaired memory and behavior. Epidemiological data indicate a potentially considerable increase in the prevalence of the disease over the next two decades (5). Most treatment strategies have been based on the cholinergic hypothesis which postulated that memory impairments in patients suffering from this disease result from a deficit of cholinergic function in brain. One of the most promising approaches for treating this disease is to enhance the acetylcholine level in the brain by means of using acetylcholinesterase (AChE) inhibitors (6). Several AChE inhibitors are being investigated for the treatment of Alzheimer. However, only tacrine, donepezil, rivastigmine, and galanthamine have been approved by the Food and Drug Administration in the United States (7). These compounds have been reported to have adverse effects including gastrointestinal disturbances and problems associated with bioavailability (8), which reinforces the interest in finding better AChE inhibitors from natural resources.

In this study, our focus was on evaluating acetylcholinesterase inhibitory properties of the essential oils obtained from dried flowers of *A. annua*, collected during different flowering phases, as there are no reports on AChE inhibitory activity of *A. annua*. We also determined the chemical composition of the essential oils by capillary gas chromatography coupled to mass spectrometry (GC–MS).

## Experimental

*Plant materials*

*A. annua* cultivar (Wuling-3938) used in this study, grew in the *Artemisiaannua* GAP Cultivation Demonstration Site of Holleypharm, Chongqing, China. The flowers were harvested at pre-flowering, full-flowering and post-flowering stages in September to November 2007. The flowers were separated from other capitula organs, leaves and stem of *A. annua*, and identified by Rongchang Luo of Holley Natural Resource Exploiture Co. Ltd, Chongqing, China and deposited in the Herbarium, College of Bioengineering, Chongqing University, Chongqing, China.

*Main instrument and reagent*

AChE, tacrine and sodium lauryl sulfate (SDS) were obtained from Sigma (St. Louis, MO). Acetylthiocholine iodide (ATCI) was obtained from FLUKA. The 5, 5-dithiobis [2-nitrobenzoic acid] (DTNB) was obtained from ACROS. All organic solvents (analytical-reagent grade) were purchased from Chongqing Chuandong Chemical Group. 0.1 molL^-1^ phosphate buffer (PB) with pH of 7.4 was used as a buffer throughout the experiment unless otherwise stated. AChE used in the assay was from electric eel (lyophilized powder, 4.392 gL^-1^ protein). The lyophilized enzyme was prepared in the buffer to obtain 2.0 mmolL^-1^ stock solution. The enzyme stock solution was kept at -20^º^C. The further enzyme-dilution was dissolved in 1.0 gL^-1 ^BSA in buffer. ATCI and DTNB were dissolved in the buffer containing 10 mmolL^-1^ and 2.5 mmolL^-1^ stock solutions, respectively.

*Essential oil extraction*

The essential oils from three samples were obtained by hydrodistillation during 6 h using a Clevenger-type apparatus (9). The yield of each essential oil was determined on average over the three replicates. These oils were dried over anhydrous sodium sulfate and kept at 4°C until analysis.

*Microplate assay for AChE activity*

The AChE inhibitory activity of esstial oils was screened by Ellman’s colorimetric method in 96-welled microplate (10, 11). Briefly, 10 µL sample, followed by adding 40 μL PB, 20 μL 2.5 mmolL^-1^ of DTNB, 10 μL enzyme solution, oscillation mixing, 37^º^C pre-incubation 10 min, then added 20 μL 10 mmolL^-1^ substrate, 37^º^C incubation 10 min, by adding 30 μL 3% of the SDS to terminate reaction. The absorbance was measured with microplate reader at 405 nm when the reaction reached the equilibrium. A control reaction was carried out using water instead of extract. The obtained absorbance value was considered 100% activity. Inhibition (%) was calculated in the following way:

I% = (100 – (Asample/Acontrol)) × 100

Where *Asample* is the absorbance of the reaction containing the extract and *Acontrol* is the absorbance of the reaction control. Tests were carried out in triplicate and a blank with phosphate buffer (PB) instead of enzyme solution was done. Extract concentration providing 50% inhibition (IC_50_) was obtained plotting the inhibition percentage against extract solution concentrations.

*Analysis of the essential oils*

GC analysations were performed using a Shimadzu GC-2010 gas chromatograph equipped with an FID and an HP-5 fused silica column (film thickness: 0.25 μm, 30 m × 0.32 mm i.d.) with a 5% phenyl-substituted methylpolysiloxane phase. The oven temperature was programmed at 40°C for 4 min and then increased to 240°C at a rate of 4°C/min. Injector and detector temperatures were 250 and 265°C, respectively. The carrier gas, helium (99.999%), was adjusted to a linear velocity of 43 cm/sec. The essential oil samples were diluted 5-fold, and 1 μL of a diluted solution was injected into the GC/MS in the split mode with a split ratio of 1/20. 

MS analyses were performed using a Shimadzu MS-QP2010 with ionization energy of 70 eV, a scan time of 0.5 s and a mass range of 33-450 amu (Atomic mass unit/Dalton (u/Da)). The components of the oil were identified by comparison of their mass spectra with those of the spectrometer database using the NIST147 mass spectral database and also with those of authentic compounds. The identifications were confirmed through comparing the fragmentation patterns and Retention index with those reported in the literature (12-14). The percentages of compounds were calculated by the area normalization method without considering response factors to establish abundances. The retention index was found with a standard mixture of C8 to C22 compounds under chromatography conditions, consistent with those of the chromatography conditions of the analyzed samples. For each essential oil, the RI and the peak area percentages were calculated as mean values of the three injections. 

## Results and Discussion

*Extraction yields*

Essential oils obtained by the conventional hydrodistillation from shade-dried flower at pre-flowering, full-flowering and post-flowering stages of *A. annua*. with 2.21%, 1.42% and 1.25% yield (w/w), respectively.

*Chemical composition of the essential oils*

In this work, the chemical composition of the three essential oil samples from A. annua was analyzed by GC–MS. Thirty-six, forty-two and thirty-nine components were identified, representing 98.88%, 99.27% and 96.57% of the total oils of the dried flowers, collected during pre-flowering, full-flowering and post-flowering phases, respectively. Table 1 depicts the compounds identification and their percentages, as well as the RI values. These values are listed in the order of their elution from HP-5ms capillary column. The main compounds in the oil of the dried flowers which were collected during the pre-flowering phase were *β*-Myrcene (37.71%), 1, 8-cineole (16.11%) and camphor (14.97%). The oil of dried flowers, collected during full-flowering phase, contained predominantly caryophyllene (19.4%), germacrene D (18.1%), camphor (15.84%), 1, 8-cineole (10.6%) and (Z)-*β*-farnesene (9.43%). 

**Table 1:** Chemical composition of the flower essential oil of *A. annua*

**RI** ^a^	**Components**	**Content (%)**		
		**A**	**B**	**C**
**816**	(3-Methyl-2-oxiranyl)methanol	-	-	0.48
**820**	2-Ethoxypropane	-	1.70	0.51
**928**	Origanene	0.25	-	-
**937**	*α*-Pinene**	0.87	-	-
**955**	Camphene	3.05	0.40	-
**976**	Sabinene	3.82	0.42	-
**981**	2,2-Dimethylhexanal	-	-	0.15
**983**	*β*-Pinene**	1.53	-	-
**991**	*β*-Myrcene	37.71	0.20	-
**995**	Yomogi alcohol	-	-	0.66
**995**	2,3-Dehydro-1,8-cineole	-	0.56	-
**1019**	(+)-4-Carene	0.13	-	-
**1021**	ND	-	0.09	-
**1029**	ND	-	0.08	-
**1032**	Limonene*	0.47	-	-
**1037**	1,8-cineole**	16.11	10.57	0.28
**1057**	Artemisia ketone	0.10	0.20	2.43
**1060**	Tricyclene	0.28	-	-
**1062**	*γ*-Terpinene	-	0.34	-
**1076**	cis-*β*-Terpineol	0.44	0.53	-
**1081**	ND	-	-	0.48
**1092**	5-(2-Methylenecyclopropyl)-1-pentanol	0.70	-	-
**1100**	(3E,5E)-2,6-Dimethyl-3, 5,7-octatrien-2-ol	3.99	1.35	2.61
**1104**	ND	-	0.23	-
**1106**	Nonanal	-	-	0.39
**1107**	Plinol C	0.59	0.63	-
**1128**	trans-p-Mentha-2,8-dienol	-	0.24	-
**1140**	ND	0.33	0.33	0.44
**1143**	Ipsdienol	-	-	0.36
**1149**	Pinocarveol	0.33	0.16	0.35
**1152**	Berbenol	0.23	-	-
**1157**	Camphor*	14.97	15.84	16.62
**1165**	Nerol	0.33	-	-
**1165**	Lavandulol	-	-	0.39
**1167**	(-)-cis-Myrtanol	-	0.22	-
**1168**	Isogeraniol	-	0.45	0.23
**1171**	ND	-	-	0.21
**1176**	Myrcenol	0.19	0.44	-
**1180**	Borneol*	0.46	1.12	3.93
**1187**	4-Terpineol	0.62	1.16	0.99
**1194**	iso-Amyl tiglate	0.47	0.48	0.36
**1199**	1, 5-Menthadien-7-ol	-	0.14	-
**1201**	*α*-Terpineol*	1.34	0.33	0.23
**1204**	Myrtenol	0.48	0.25	-
**1217**	trans-3(10)-Caren-2-ol	0.25	0.29	0.51
**1234**	(E)-3(10)-Caren-4-ol	-	0.20	-
**1247**	(2E)-2,7-Dimethyl-2,6-octadien-1-ol	0.10	0.15	-
**1248**	ND	-	-	0.20
**1259**	4,6,6-Trimethylbicyclo[3.1.1]hept-3-en-2-yl acetate	-	1.60	-
**1279**	Nerol acetate	0.21	-	0.42
**1310**	Hydroxy-*α*-terpenyl acetate	-	0.57	-
**1377**	Copaene	-	1.09	1.44
**1392**	*β*-Elemen	-	-	1.46
**1420**	*β*-Caryophyllene**	2.32	19.41	16.27
**1445**	*β*-Farnesene	2.57	9.43	9.05
**1454**	*α*-Caryophyllene	-	1.05	-
**1470**	Chamigren	1.76	-	-
**1479**	Germacrene D	1.90	18.13	3.96
**1495**	*γ*-Elemene	0.31	-	-
**1498**	Germacrene B	-	0.88	-
**1570**	(-)-Spathulenol	-	1.81	7.21
**1575**	*β*-Caryophyllene oxide*	-	2.99	15.84
**1681**	Aromadendrene oxide-(2)	-	2.85	2.22
**1904**	ND	-	-	1.35
**1907**	*δ*-Cadinol	-	1.09	-
**1919**	(10Z,12Z)-9-Methyl-10,12-hexadecadienyl acetate	-	-	1.13
**1942**	ND	0.45	-	-
**1961**	ND	-	-	0.75
**1967**	*n*-Hexadecanoic acid	-	-	4.37
**1977**	ND	0.34	-	-
**1979**	9,12,15-Octadecatrienal	-	-	0.26
**2106**	trans-Phytol	-	-	0.37
**2131**	Stearolic acid	-	-	0.47
**2297**	2,6,10,14-Tetramethylheptadecane	-	-	0.62
**Total identified**		98.88	99.27	96.57
**Monoterpenes**		89.11	36.95	29.59
**Sesquiterpenes**		8.86	58.73	57.45
**Fatty acids and aliphatic esters**		-	-	7.22

RI^a^: The Retention index relative to C8-C22 *n*-alkanes on the HP-5ms column; ND: Not identified; (-): Not detected; A: Pre-flowering oil; B: Full flowering oil; C: post-flowering oil

**Figure 1:**
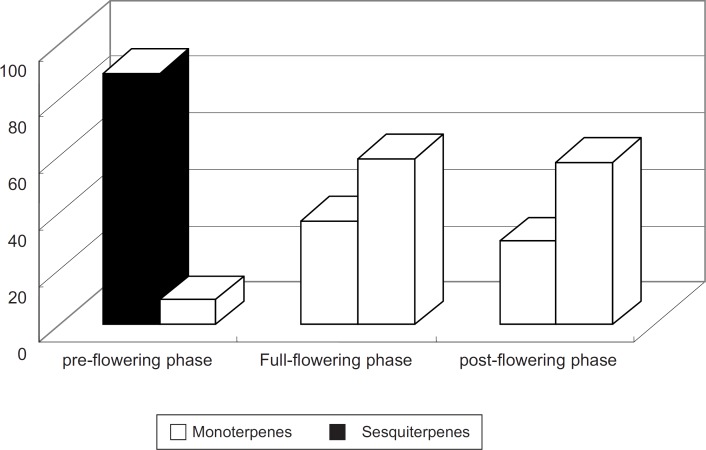
The change of the content of the monoterpenes and sesquiterpenes from pre-flowering to post-flowering stage

The major constituents which were identified in the oil of dried flowers and were collected during the post-flowering phase were camphor (16.62%), caryophyllene (16.27%), *β*-caryophyllene oxide (15.84%), *β*-farnesene (9.05%) and (-)-spathulenol (7.21%). All of these constituents, *i.e.* camphor, 1, 8-cineole, caryophyllene, *β*-caryophyllene oxide, *β*-farnesene and (-)-spathulenol, have already been reported in the oil of *A. annua*from different location (4). The monoterpenes and sesquiterpenes are the major properties of the oils of dried flowers. But the content of the monoterpenes and sesquiterpenes has markable changes from pre-flowering to post-flowering stage (Figure 1).

As far as whole flowering phase is concurred, the monoterpenes decreased from 89.11% to 29.59%, while the sesquiterpenes increased from 8.86% to 58.73%. Other compounds *i.e.*fatty acids and aliphatic esters which were also detected in the oil of flowers, collected during the post-flowering phase.

*Acetylcholinesterase inhibitory activity*

Acetylcholine is a compound liberated at the synaptic gap as a neurotransmitter. Neurotransmitter disturbances and insufficient cholinergic functions are identified among the pathological features in central nervous system disorders. The most important changes observed in the brain are a decrease in cortical levels of the neurotransmitter acetylcholine. Therefore,inhibition of acetylcholinesterase can restore the level of acetylcholine in the brain. Plants have been used traditionally to enhance cognitive function and to alleviate other symptoms associated nowadays with Alzheimer’s disease (15). Most of the drugs used in Alzheimer therapy are formed by an enzyme inhibitor,*e.g*. galantamine, isolated from the extract of snowdrop (8). Few reports exist for the inhibitor activity of acetylcholinesterase by essential oils. The AChE inhibitory activity of the essential oils of dried flowers *A. annua* has never been reported in the past. Essential oil of this plant was tested to determine their ability as acetylcholinesterase inhibitors and the results are depicted in Table 2. 

**Table 2:** Acetylcholinesterase inhibition capacity represented by IC_50_ (mg mL^-1^) ^a^, of essential oils

**Pre-flowering oil**	**Full-flowering oil**	**Post-flowering oil**	**Tacrine**
1.25 ± 0.09	2.92 ± 0.16	0.13 ± 0.02	5.0 × 10^-5^

^a ^: Averages ± SD were obtained from three different experiments

The greatest inhibitory activity was exhibited by the essential oil of flowers of the plant collected from the post-flowering phase (IC_50_ = 0.13 ± 0.02 mgmL^-1^). Analysis of the results shows that these oils are moderate AChE inhibitors. Galantamine, a compound used pharmacologically, showed an IC_50_ value of 1 mg/mL (16).

In previous reports, it has been mentioned that 1,8-cineole, camphor, *α*-pinene, -pinene, borneol, linalool, bornyl acetate, linalyl acetate, menthone, carvone, anetole, anisole, eugenol, nonyl alcohol, isomenthol, (-)-menthol, (+)-menthol, citronellol, *β*-myrcene, terpinene, 3-carene, *β*-caryophyllene and *β*-caryophyllene oxide have anti-AChE activity (17-20). It was reported that 1, 8-cineole /*α*-pinene and 1, 8-cineole/caryophyllene oxide combinations were minor synergy. In contrast, a combination of camphor and 1, 8-cineole was antagonistic. This study shows that the high concentration of 1, 8-cineole and the low concentration of camphor in the oil may result in an increase in its anticholinesterase activity (17).

The anti-AChE activity of the oil of *A. annua* flower is mainly attributed to *α*-Pinene, *β*-Pinene, Limonene, 1, 8-cineole, Camphor, Borneol, *α*-Terpineol, *β*-Caryophyllene and *β*-Caryophyllene oxide. The different anti-AChE activity of the flower oil of *A. annua* at three flowering stages may have resulted from the different content of those terpenoids and their different interactions with anti-AChE activity.The synergy of 1, 8-cineole/*α*-pinene and the antagonism of 1, 8-cineole /camphor coexist in the pre-flowering oil. The synergy of antagonistic 1, 8-cineole/ caryophyllene oxide and the antagonistic action of 1, 8-cineole /camphor still coexist in the full- and post-flowering oil. 
